# Obliquity Control On Southern Hemisphere Climate During The Last Glacial

**DOI:** 10.1038/srep11673

**Published:** 2015-06-26

**Authors:** C.J. Fogwill, C.S.M. Turney, D.K. Hutchinson, A.S. Taschetto, M.H. England

**Affiliations:** 1 Climate Change Research Centre School of Biological, Earth and Environmental Sciences, University of New South Wales, Sydney, NSW 2052. Australia; 2 ARC Centre of Excellence for Climate System Science, Australia

## Abstract

Recent paleoclimate reconstructions have challenged the traditional view that Northern Hemisphere insolation and associated feedbacks drove synchronous global climate and ice-sheet volume during the last glacial cycle. Here we focus on the response of the Patagonian Ice Sheet, and demonstrate that its maximum expansion culminated at 28,400 ± 500 years before present (28.4 ± 0.5 ka), more than 5,000 years before the minima in 65°N summer insolation and the formally-defined Last Glacial Maximum (LGM) at 21,000 ± 2,000 years before present. To investigate the potential drivers of this early LGM (eLGM), we simulate the effects of orbital changes using a suite of climate models incorporating prescribed and evolving sea-ice anomalies. Our analyses suggest that Antarctic sea-ice expansion at 28.5 ka altered the location and intensity of the Southern Hemisphere storm track, triggering regional cooling over Patagonia of 5°C that extends across the wider mid-southern latitudes. In contrast, at the LGM, continued sea-ice expansion reduced regional temperature and precipitation further, effectively starving the ice sheet and resulting in reduced glacial expansion. Our findings highlight the dominant role that orbital changes can play in driving Southern Hemisphere glacial climate via the sensitivity of mid-latitude regions to changes in Antarctic sea-ice extent.

The Last Glacial Maximum (LGM) – traditionally defined from sea level records as having taken place between 23,000 and 19,000 calendar years ago – was apparently driven by insolation changes at 65°N^1^,^2^, and reflects the period when the world’s ice sheets reached their maximum extents, locking up the greatest volume of ocean water^1^. However, as sea level is an integrated signal, it cannot be used to distinguish globally synchronous ice-sheet maxima from the variable regional ice sheet maxima that may provide critical insights into the mechanisms of regional and hemispheric climate change.

The timing of glacial advance is complicated in the mid-latitudes of the Southern Hemisphere (SH), where ice fields and glaciers appear to have reached their maximum extent prior to the global LGM, suggesting sensitivity to forcing mechanisms other than Northern Hemisphere insolation^3^,^4^,^5^,^6^,^7^. Given the latitudes of glaciers and ice in the Southern Hemisphere and the relationship between high insolation and ice ablation rates, it is counterintuitive that a northern signal could have played such a dominant role^8^. This has been highlighted by recent analysis from the WAIS divide ice core in West Antarctica, which implies a far more active role for regional orbital forcing in driving Termination 1^9^. This has also been supported by recent climate modeling studies^7^, raising the question as to whether such change may have also driven the onset of the early LGM (eLGM) defined from palaeoecological studies at ~28.5 ka in the mid- to high-latitude Southern Hemisphere^4^,^10^. As such, resolving the timing of variability in regional ice-sheet maxima is key to understanding global climate forcing, as well as establishing possible ice-sheet climate feedbacks.

Here we focus on the timing of maximum ice-sheet expansion in Patagonia, southernmost South America, and across the wider south Pacific region, where paleo-environmental, radiocarbon and relative sea level constraints strongly support a strong eLGM signal^4^,^6^,^11^ (Fig. 1). Whilst evidence of an eLGM response is not apparent in all paleoclimate records^12^,^13^, this may reflect either that reconstructing palaeoenvironemnetal change from proxy evidence may be extremely challenging in certain environments^12^, or perhaps that the ocean-atmosphere systems may not parallel one another under glacial conditions^14^. As such, direct dating of these events has proved problematic, due to relatively large uncertainties over both ^14^C calibration^15^, and proxy interpretations^12^, making the identification of leads and lags in the global climate system challenging. Cosmogenic exposure analysis performed on glacial erratics and landforms of former glaciers and ice sheets offers an alternative and independent method by which to directly constrain the timing of maximum ice advance^16^; however to date this has also failed to resolve the debate fully in the Southern Hemisphere due to similarly large uncertainties, in this instance associated with the globally integrated production rate for cosmogenic isotope production^16^.

Here we recast previously published cosmogenic isotope studies from a series of glacial advances of the Patagonian Ice Sheet that spans more than ten degrees of latitude^11^,^17^,^18^,^19^,^20^,^21^,^22^, and investigate the potential drivers of a possible eLGM using a suite of climate models incorporating prescribed and evolving sea-ice anomalies.

## Results and discussion

We apply the production rate derived in New Zealand^23^; which is statistically indistinguishable from that derived from southernmost South America^24^; to a series of glacial advances of the Patagonian Ice Sheet during Oxygen Isotope Stage 2. Taken together, we derive a statistically indistinguishable eLGM advance across the entire Patagonian Ice Sheet, centered on 28,420 ± 460 years (see materials and methods for detailed description). This age is statistically different to the global LGM (23,000-19,000 years), and indicates the maximum extent of the Patagonian Ice Sheet was achieved over 5,500 years before the global LGM; this is consistent with estimates of maximum cooling in New Zealand at 28,500–30,500 years ago^4^,^6^, suggesting a different regional forcing mechanism drove maximum ice sheet expansion across Southern Patagonia than insolation changes at 65°N.

To investigate the mechanisms that drove this apparent early eLGM, we simulate the effects of orbital changes using a suite of climate models incorporating prescribed and evolving sea-ice anomalies. Previous modeling studies have argued that sea ice plays a critical role in atmospheric circulation change in the Southern Hemisphere, and can significantly alter the location and intensity of the Southern Hemisphere jet stream^7^,^25^. However, these studies either integrate changes over long time periods, or prescribe the extent of sea ice in an atmospheric general circulation model, thus they may not fully resolve the detail of potential changes in ocean properties that can lead to important feedbacks within the climate system. Therefore, given the apparent sensitivity of sea-ice extent to both CO_2_ and obliquity, we use a coupled ocean-atmosphere-land-sea-ice climate model^26^ to examine in detail how sea ice evolves in a simulation forced under eLGM conditions.

**Figure 1:**
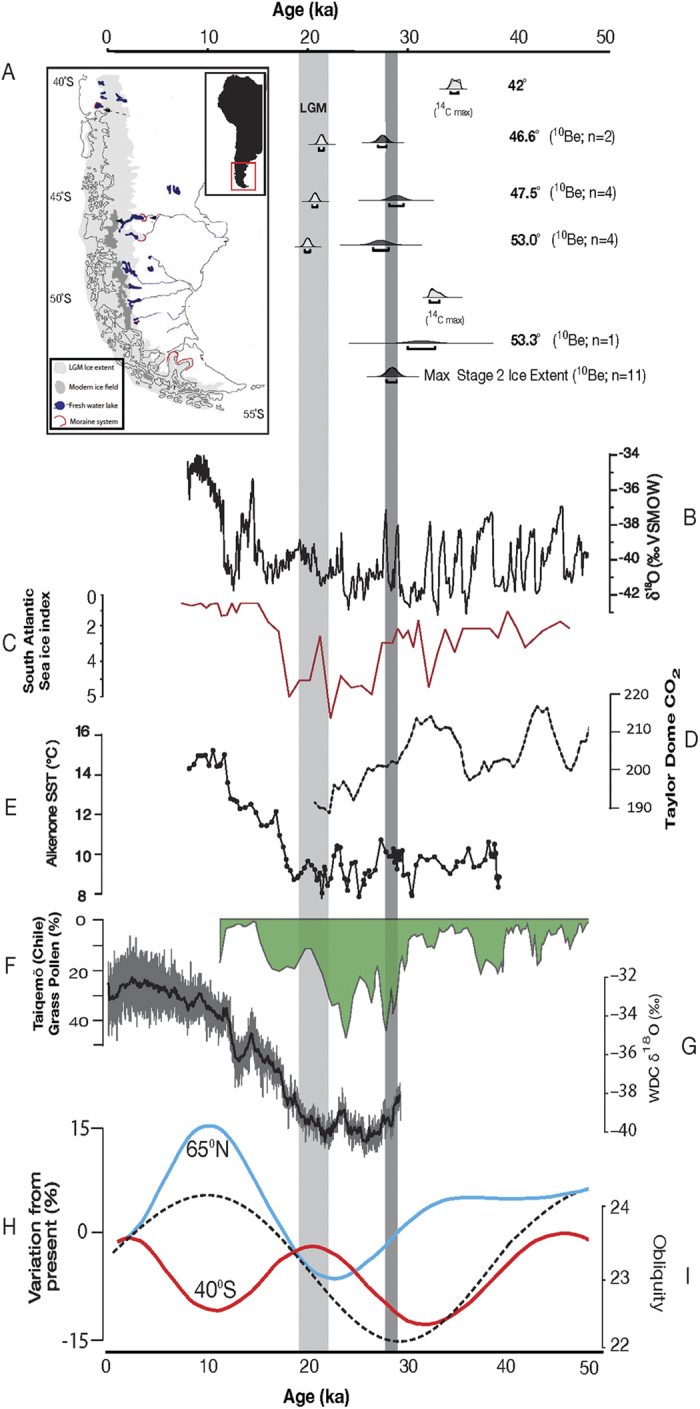
A . Outline map of southern most South America (created in Adobe Illustrator CS5 v15.0.0), showing the proposed extent of the LGM^37^, and the location and distribution of the modern Patagonian ice fields. Cosmogenic ages and constraining data for maximum Patagonian Ice Sheet extent and orbital changes over the last 50 ka. Cosmogenic exposure ages are calculated using the Cronus online calculator version 2.2.1 using the production rate recently derived in the Maculay Valley in the Southern Alps of New Zealand (P_NZ_)^16^,^23^. Light gray column denotes defined age of ‘Last Glacial Maximum’; dark gray denotes Patagonian 1σ range. **B**. Oxygen isotope record of the GISP2 ice core, Greenland. **C**. Diatom-based sea ice reconstruction from South Atlantic core TN 057-13^30^. **D**. Taylor Dome CO_2_^43^. **E**. Alkenone SST record from ODP Site 1233^44^. **F**. Taiquemo´ (HE94-2B) grass pollen curve for the past 40 kyr^5^. **G.** Water isotope ratios from WAIS Divide Ice Core^9^. **H**. Plot of variation in mean summer insolation at 40**°**S and 65**°**N compared to present (data is archived at the World Data Center for Paleoclimatology, Boulder, Colorado, USA. http://www.ncdc.noaa.gov/paleo/forcing.html). **I**. Obliquity curve.

**Figure 2:**
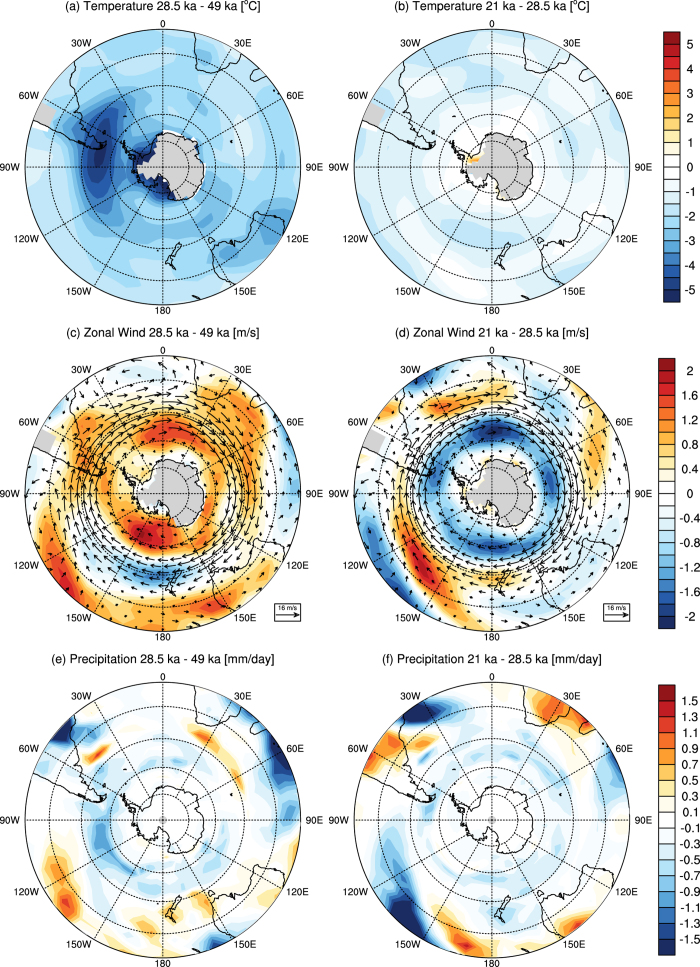
CSIRO Mk3L model simulations for austral summer average taken from January to March of (**a**, **b**) 850 hPa temperature differences; (**c**, **d**) 850 hPa zonal wind differences and (**e**, **f**) precipitation differences between the 28.5 ka minus the 49 ka experiments (left), the 21 ka minus the 28.5 ka experiments and (right). Each average is taken from the last 50 years of the experim+ents (created in NCAR Command Language).

**Figure 3:**
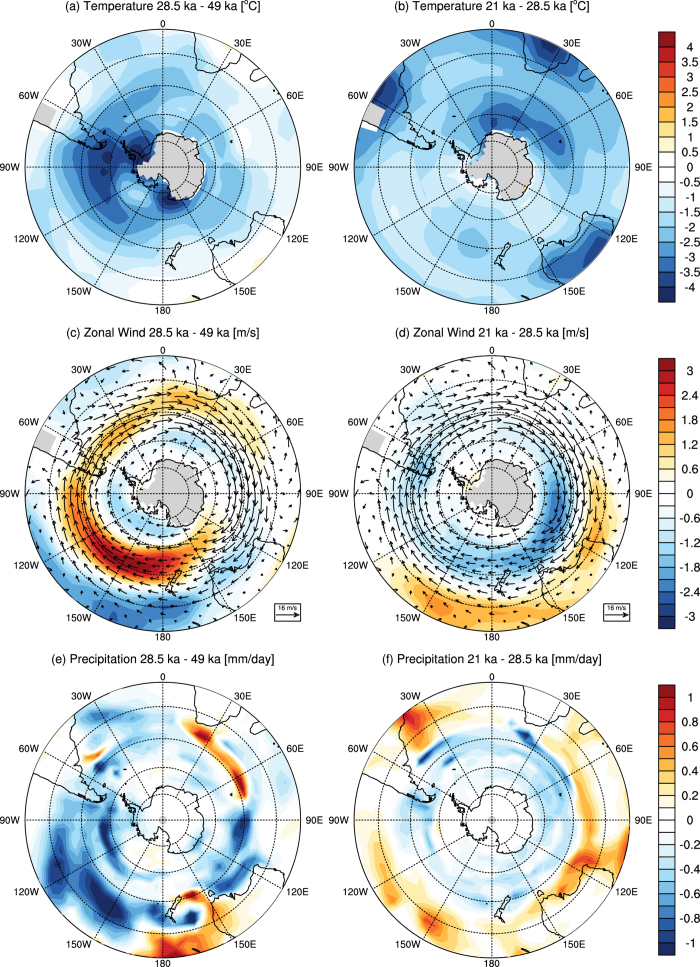
CSIRO Mk3L simulations of austral winter average taken from July to September of (**a**,**b**) 850 hPa temperature differences; (**c**,**d**) 850 hPa zonal wind differences and (**e**,**f**) precipitation differences taken from the 21 ka minus the 28.5 ka minus the 49 ka experiments (left), the 21 ka minus the 28.5 ka experiments and (right). Each average is taken from the last 50 years of the experiments (created in Adobe Illustrator CS5 v15.0.0).

**Figure 4:**
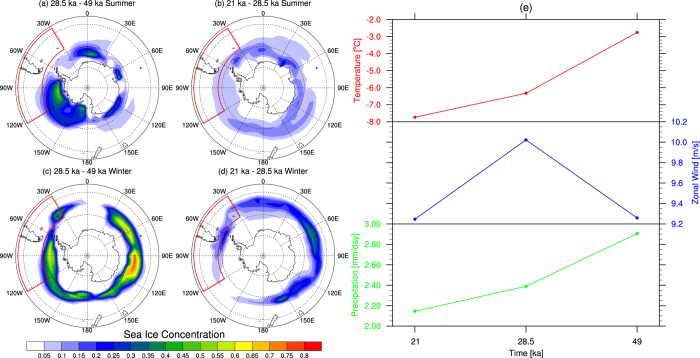
CSIRO Mk3L model simulations for sea ice concentration difference during (**a**-**b**) austral summer (January to March) and austral winter (July to September) for the (**a**-**c**) 28.5 ka minus the 49 ka experiment and the (**b**-**d**) 21 ka minus the 28.5 ka experiment and (**e**) the 50 year annual averages in the region bounded by 41°S to 57°S, and 121°W to 31°W for 850 hPa temperature, 850 hPa zonal wind and precipitation (created in NCAR Command Language).

**Figure 5:**
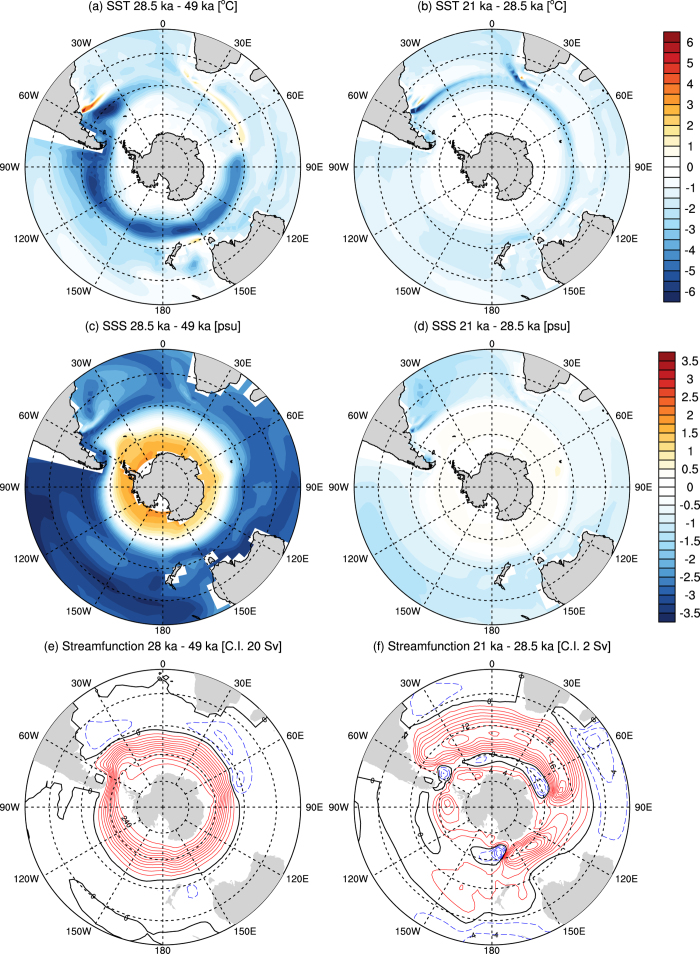
CSIRO Mk3L model simulations of annual average (**a**,**b**) sea surface temperature (SST) differences and (**c**,**d**) sea surface salinity (SSS) and (**e**,**f**) barotropic streamfunction. Differences between the 28.5 ka minus the 49 ka experiments (left), the 21 ka minus the 28.5 ka experiments and (right). Each average is taken from the last 50 years of the experiments (NCAR Command Language).

We simulate a suite of key time slices: (i) the Last Glacial Maximum, centered on 21 ka, (ii) the obliquity minimum centered on 28.5 ka, (iii) the obliquity maximum centered on 49 ka, and (iv) the pre-industrial climate based on year 1780 parameters as the control simulation (Table S2). The 50-year average austral summer (January to March) and winter (July to September) temperatures, zonal wind, precipitation and sea ice differences between our simulations can be seen in Figs 2, 3, 4, 5. We present full circumpolar plots to allow inter-comparison with both the key proxy records and each of the variables across the Southern Hemisphere (Fig. 1).

Our simulations identify a marked reduction in temperature during 28.5 ka relative to 49 ka over Patagonia during the austral summer in Fig. 2a, and winter in Fig. 3a. Whilst the region of cooling varies spatially between seasons its focus extends from 40**°**S to beyond 60**°**S and is centered on 53**°**S, covering a broad longitudinal swathe of the region (120 to 30**°**W). This temperature reduction is accompanied by a marked increase in wind strength, implying a notable shift in Southern Hemisphere atmospheric circulation between maximum and minimum obliquity. Our simulations suggest that during the austral summer the westerly winds intensify and broaden hemisphere wide, only reducing marginally over New Zealand (Fig. 2c). This is consistent with changes to minimum obliquity, which reduces summer-winter seasonality. The result for the austral summer is a change in the vertical and meridional distribution of the mean flow such that it weakens the seasonal migration of the Hadley Cell in the Southern Hemisphere, leading to a northward shifted subtropical jet during the 28.5 ka period relative to mean circulation at 49 ka. In the austral winter the flow differences between the 28.5 ka and 49 ka simulations show a more zonal pattern (Fig. 3c).

At the same time, the orbital parameters and greenhouse gas concentration at 28.5 ka drive an asymmetric equatorward expansion of Antarctic sea ice (Fig. 4a) that intensifies the southern mid-latitude sea surface temperature (SST) gradient (Fig. 5a). It has been previously shown that changes in sea-ice extent and mid-latitude SST gradients affect the location of storm tracks and the eddy-driven jet through increases in atmospheric baroclinicity^25^,^27^. During the 28.5 ka period, the strengthened SST gradient in the southern mid-latitudes intensifies the lower troposphere baroclinicity, leading to a southward shift of the eddy-driven jet. This change leads to a broadening of the latitudinal zone of the storm-tracks that in turn intensifies the westerlies, with a marked focus over southern South America (Fig. 2c). It appears, therefore, that the stronger winds and cooler temperatures at 28.5 ka nourished the Patagonian ice sheet more effectively at 28.5 ka than during the subsequent global LGM at 21 ka.

The increase in the westerly winds is seen year-round, despite variations in structure, with expansion in the austral summer months and strong zonation in winter (Figs 2c and 3c) During the 28.5 ka austral winter, the Hadley Cell slightly weakens as a consequence of the reduced seasonality in a lower obliquity climate. However, the largest changes in the zonal mean flow distribution occur in the strengthening of the Southern Hemisphere storm track. The expansion of the Antarctic sea ice to the southern margin of the lower tropospheric baroclinic zone acts to increase the mid-latitude temperature gradient, thus intensifying baroclinicity from 60**°**S to 45**°**S at 28.5 ka. The resulting strengthened westerlies have a particularly marked effect in the Pacific sector where sea ice reaches its most northward latitude (Fig. 4a). This result differs somewhat in extent from recent simulations using a coupled general circulation model of intermediate complexity which posit maximum temperature changes were more zonal and concentrated at around 55**°**S^7^.

The expansion of the Antarctic sea ice during 28.5 ka compared to 49 ka maximum obliquity period directly affects the Southern Ocean, as seen in the reductions predicted in SST Figure (5a). Sea surface salinities increase south of Drake Passage, and decrease to the north of this region (Fig. 5c). This pattern of salinity forcing increases the density gradient across the Antarctic Circumpolar Current (ACC), significantly enhancing ACC transport through the thermal wind balance. The meridional expansion of sea-ice at 28.5 ka increases the SST gradient at mid-latitudes, particularly during austral summer when sea ice experiences larger changes compared to the 49 ka model runs.

Our simulations suggest that low obliquity triggered marked intensification of atmospheric circulation in the SH, resulting in a significant increase in the Southern Ocean barotropic streamfunction between 49 ka and 28.5 ka (Fig. 5e). Although this trend continued into the global LGM (Fig. 5f) –agreeing with previous LGM modeling studies^28^– the regional year-round cooling over Patagonia at 28.5 ka led to cool winters and critically cool summers, with only a slight change in precipitation, encouraging positive ice-sheet mass balance during the obliquity minimum. These conditions could have driven marked ice-sheet expansion across Patagonia at 28.5 ka through positive mass balance with low rates of summer ablation.

The mechanisms behind the pronounced atmospheric cooling driven by expansion of Antarctic sea ice and orbital parameters can be explored through an independent suite of numerical experiments performed with the atmospheric component of the NCAR CCSM3 model (See methods)^29^. The model simulated the meridional shift of the Hadley cell to the 28.5 ka orbital conditions and the intensification of the Southern Hemisphere storm track caused by the northward expansion of Antarctic sea-ice, supporting the CSIRO Mk3L model simulations. Whilst in this case the use of an atmospheric model limits the realistic representation of past climate periods due to the lack of an ocean feedback, it supports our interpretation of the atmospheric response to orbital forcing, related to a prescribed change in sea-ice extent (Figure S1).

Our simulations demonstrate that while cooling was focused over Patagonia, the atmospheric conditions exhibit an influence across a broad swathe of the mid- to high-latitudes of the SH in our fully coupled model simulations. The relationship with sea ice, air temperature and precipitation is key to understanding the ice-sheet response. Our results suggest that the regional year-round cooling over Patagonia led to cool winters and cool summers, with only slight changes in precipitation, encouraging positive ice-sheet mass balance during the obliquity minimum. These conditions would have driven marked ice-sheet expansion across Patagonia at 28.5 ka through positive mass balance with low rates of summer ice ablation despite relatively high CO_2_ when compared to 21 ka (Fig. 1d). Although the relationship with precipitation is spatially variable between the simulations, a comparison between the 50-year mean values shows a stepwise downward trajectory in precipitation between our simulations across the region (Fig. 4e). We suggest this downward trend reflects the combination of predicted temperature and precipitation decreases and the increase in sea-ice extent between 28.5 ka and 21 ka (Fig. 4b,d,e). At 21 ka our model results implys sea-ice extent is greater year-round having the net effect of reducing regional temperatures and effective precipitation in the region during both the austral summer and the austral winter (Fig. 4e). Our interpretation is supported by key palaeoclimate records^4^,^5^,^6^,^30^ (Fig. 1C–F), and agree broadly with recent climate modelling simulations^7^, there are key differences that must be addressed. These differences largely reflect the interpretations of palaeoenvironmental records from New Zealand^13^ and contrasting interpretations from records of sea ice expansion in different sectors of the Southern Ocean^12^,^30^. These differences are addressed in turn.

Firstly, cosmogenic isotope analysis from glacial moraines from Lake Pukkaki, New Zealand appear to show significant expansion of ice at around 42 ka BP, which is more extensive than that recorded between 28-25 ka suggesting an early advance within this glacial catchment during a period of high obliquity^13^. Although this is a well dated example of potentially high obliquity driven glacier advance, it is not apparent in any other regional palaeoenvironmental records^4^, or indeed in other proximal glacial systems in New Zealand^6^,^31^, and could therefore potentially reflect the influence of site specific topographic, tectonic or possible geomorphic factors^32^. Additionally emerging data suggests there were multiple expansions of ice at some sites in New Zealand and potentially in Chile during Oxygen Isotope Stage 3^31^,^33^. Although we find no evidence of them at the sites highlighted in this study, we cannot discount the possibility of earlier glacial expansions that were subsequently overrun by the eLGM expansion we report here. Clearly more detailed direct chronologies are required to fully resolve this question. Secondly, it is apparent that sea ice proxy reconstructions from marine cores in the South Atlantic and Scotia Sea provide very different interpretations across the mid- to high-latitude Southern Ocean during the last 50,000 yrs^12^,^30^. Whilst our simulations apparently agree with the reconstruction of South Atlantic sea ice^30^, they contrast with that derived from marine cores in the Scotia Sea^12^. These differences may well reflect in part the challenges of reconstructing sea ice cover from proxy evidence with limited age control. This has been highlighted by many studies, particularly in the Arctic, where even Holocene studies, provide contrasting interpretations of sea ice concentration^34^. This, however, does not invalidate our interpretation of either the timing or mechanism we propose which is supported by multiple lines of palaeoenvironmental evidence^4^,^6^,^35^.

## Conclusions

In order to explain the apparent eLGM cooling in the Southern Hemisphere mid-latitude relative to the Northern Hemisphere, recent studies have suggested that regional insolation variations may play a greater role in climate change than previously supposed^4^,^9^. In light of our reanalysis of geological data and independent model simulations, we demonstrate that the early regional maximum across Patagonia at 28.5 ka may have been driven by the effects of changing obliquity, which triggered marked changes across the broader Southern Hemisphere atmospheric circulation, with cold conditions year-round in the southern mid-latitudes. While sea-ice expansion was notable, intense increases and seasonal zonation of the Southern Hemisphere wind belts restricted northward expansion of sea ice across the Drake Passage. This contrasts with conditions at 21 ka, when sea-ice extent increased year-round, extending to 53**°**S even in summer months (Fig. 4). Reduced precipitation relative to 28.5 ka appears to account for the subsequent smaller glacial expansions during the global LGM. The latter is evidenced by the moraine complexes across Patagonia, which took place against rising obliquity and increasing Antarctic sea-ice cover^36^, resulting in ever-decreasing amounts of snowfall, consistent with the observed smaller advances.

The mechanism we propose implies that sea ice changes driven by Southern Hemisphere insolation changes played a significant role in driving climate regionally and in the ultimate development of the global LGM. Our model simulations provide an explicit mechanistic link between regional climate, sea ice expansion and changes in meridional temperature gradients that explains how obliquity-driven regional insolation played a dominant role in the growth of ice masses across the mid-latitude Southern Hemisphere. It is clear that Southern Hemisphere ice-sheet growth does not agree with the accepted paradigm that global ice extent is driven by insolation changes at 65°N, resolving a long standing question and demonstrating the important role sea ice plays in modulating Southern Hemisphere climate, which has implications for future climate prediction.

## Materials and Methods

Here we apply the production rate derived in New Zealand^23^; which is statistically indistinguishable from that derived from southernmost South America^24^; to previously published cosmogenic isotope studies from a series of glacial advances of the Patagonian Ice Sheet during Oxygen Isotope Stage 2 (Fig. 1A)^11^,^19^,^20^,^21^,^22^,^23^. By incorporating data from each of these studies, the sites span a large latitudinal range extending from 42°S to 53°S. This range covers a significant proportion of the former Patagonian Ice Sheet, which expanded and coalesced at the LGM to form a continuous ice mass from 35°S to 56°S^37^ (Table S1). Following the approach taken in North America^38^, we identify inner and outer maximum ‘LGM’ constraints that effectively bracket the direct dating of the maximum LGM advance in Patagonia from cosmogenic exposure analysis. These independently derived bracketing radiocarbon chronologies record the maximum Stage 2 ice advance at key localities at the northern and southern limits of our latitudinal transect (See Table S1 for details). To asses the true age of the LGM maximum ice sheet advance using cosmogenic isotope analysis we follow the approach that the oldest ages from any one advance provides the closest estimate of its true age^39^,^40^. Thus we reject significant outliers that in this environment, most probably reflect the effect of downwasting of the moraine and subsequent exposure of erratics^39^, and accept that the mean ages of the statistically-oldest estimates at each site provide a precise chronological framework for these glacial advances^11^,^17^,^18^,^19^,^20^,^21^,^22^ (see Table S1). To avoid the well reported problem of ‘suck in and smear’^41^ from the multiple radiocarbon chronologies available, we choose to reject many of the numerous potential radiocarbon constraints from the Chilean Lake District and the Magellan Region due to the possible inclusion of older derived organic material and sample reproducibility^11^,^42^. Finally, we use well-dated inner Fenix IV moraines from Lago Buenos Aires at 46.6°S to provide an important minimum constraint on the timing of maximum LGM ice-sheet expansion^20^. We define the timing of the maximum ice sheet expansion through the application of Bayesian statistical analysis, using the *C_Combine* function in the dating program OxCal (http://c14.arch.ox.ac.uk), allowing us to test for the synchroneity of maximum ice extent in South America as recorded by the outermost moraines at each of the sites in order to compare with potential forcing mechanisms.

To gain insights into the potential drivers of the eLGM in Patagonia and across the broader Southern Hemisphere we examine the response of the atmosphere, ocean and sea ice to changes in orbital forcing parameters and greenhouse gas concentrations using a fully coupled climate model CSIRO Mk3L. We also examined sensitivity experiments using NCAR CAM3. The climate modeling experiments presented here do not provide a full reconstruction of these past climates; rather they are designed as sensitivity experiments to assess the mechanism of obliquity forcing upon climate as proposed in this paper. The full details of these simulations are outlined in detail in the Supplementary Material.

## Additional Information

**How to cite this article**: Fogwill, C. J. *et al.* Obliquity Control On Southern Hemisphere Climate During The Last Glacial. *Sci. Rep.* **5**, 11673; doi: 10.1038/srep11673 (2015).

## Supplementary Material

Supplementary Information
