# Supplementary material for: Obliquity Control On Southern Hemisphere Climate During The Last Glacial
Source: Sci Rep. 2015 Jun 26;5:11673. doi: 10.1038/srep11673 (PMC4650511; doi:10.1038/srep11673)
Supplement: Supplementary Information [file srep11673-s1.doc]

**obliqUity control on southern hemisphere climate during the last glacial**

C.J. Fogwill *^1^, C.S.M. Turney^1^, D.K. Hutchinson^1,2^, A.S. Taschetto^1,2^, M.H. England^1,2^

^1^Climate Change Research Centre School of Biological, Earth and Environmental Sciences, University of New South Wales, Sydney, NSW 2052. Australia

^2^ARC Centre of Excellence for Climate System Science, Australia

*Corresponding author: email [c.fogwill](mailto:c.fogwill@unsw.edu.au)@unsw.edu.au

Tel: +61 2 93859766 Fax: +61 2 9385 8969

**Supplementary Information**

**Chronological Constraints**

Previous studies applying cosmogenic exposure analysis in Patagonia have had recourse to use the globally averaged production rate in the absence of a locally derived production rate site. Recently, however, using independent radiocarbon control combined with high precision ^10^Be cosmogenic isotope analysis, a local production rate for Patagonia has been derived[^1^](#_ENREF_1) that overlaps at 1σ with the production rate recently derived in the Southern Alps of New Zealand (P_NZ_)[^2^](#_ENREF_2). The upper limit of this range is more than 12% below the average global production rate, when calculated using the same scaling method reported by Balco et al.[^3^](#_ENREF_3) Given the geographic location of the site used to generate the Patagonian production rate (Lago Argentino, 50**°**S) is at a similar altitude and latitude as many of the key locations that suggest a regional eLGM, it is now possible to robustly test ^10^Be exposure chronologies of ice margin change against independent records of climate change regionally and globally.

Table S1 outlines the recast cosmogenic ^10^Be data set recording the timing of the maximum ice sheet expansion based upon multiple cosmogenic nuclide studies over the latitudinal transect. The ages have been recast in light of new calibrations sites[^1-3^](#_ENREF_1), updated geomagnetic models and muon considerations[^3^](#_ENREF_3). It also allows us to take account of refinements to the ^10^Be half life and ^10^Be AMS standards that have been highlighted since the publication of these constraints[^4^](#_ENREF_4). The age calculations are using the Cronus On-line calculator, using version 2.2.1[^3^](#_ENREF_3), using the Macaulay Valley P_NZ_ production rate[^2^](#_ENREF_2) (available at http://hess.ess.washington .edu/math/al_be_ v22/Age_input_NZ_calib.html).

In order to bracket the direct dating of the maximum LGM advance we use independently derived maximum radiocarbon constraints on the age of Stage 2 ice advance from two key localities at the northern and southern limits the latitudinal transect. Firstly at 42˚S in the Chilean Lake District, organic material overlain by diamicts provides a maximum constraint on the age of ice advance, which is calibrated here using the IntCal09 dataset[^5^](#_ENREF_5) records a range of 34,300-33,530 cal yrs BP (mean 33,900 cal yrs BP)[^6^](#_ENREF_6). We acknowledge there are many other radiocarbon constraints within this data set, however, we chose to focus on this constraint due to its narrow range and good reproducibility in comparison to many in the region. A second key constraint comes from 53˚S in the Strait of Magellan, at Rio Tres Brazos, where shells within a diamict provide another independent maximum constraint on ice sheet advance that has a calibrated range of 32,420-31,460 cal yrs BP (mean 31,950 cal yrs BP)[^7^](#_ENREF_7). We further support our interpretation of the timing of maximum ice sheet expansion with a minimum constraint provided by the cosmogenic ages obtained on the Fenix III moraine in Lago Buenos Aires at 46.6˚S[^8^](#_ENREF_8), the third limit from Lago Pueyrredon at 47.5˚S[^9^](#_ENREF_9) and the frontal moraines in the Straits of Magellan at 52.9˚S[^10^](#_ENREF_10) (Figure 2). These moraines important constraints on the timing of maximum LGM Patagonian ice sheet advance between 20,000-22,000 yrs, and follows the approach taken in North America, identifying an inner ‘maximum’ LGM advance[^11^](#_ENREF_11).

To asses the true age of the maximum LGM ice sheet advance using cosmogenic isotope analysis we follow the approach that the oldest ages from each advance provides the closest estimate of the true age of the moraine[^12^](#_ENREF_12). Thus, we reject significant outliers. A Bayesian modelling approach using the *C_Combine* function was undertaken in OxCal ([http://c14.arch.ox.ac.uk](http://c14.arch.ox.ac.uk/)) it is possible to identify significant outliers that in these environments. We suggest that these most probably reflect the effects of downwasting of the moraine and subsequent exposure of erratics[^12^](#_ENREF_12). By rejecting these, it is possible to derive a precise chronological framework for the different glacial advances at each site and a statistically-coherent mean age estimate of maximum ice extent (see Table 1). The resulting age models for these sites provide an opportunity to test for the synchroneity of maximum ice extent in South America as recorded by the outermost moraines at each of the sites.

**Earth system modeling experiments**

To gain insights into the potential drivers of the eLGM in Patagonia and across the broader Southern Hemisphere we examine the response of the atmosphere, ocean and sea ice to changes in orbital forcing parameters and greenhouse gas concentrations using a fully coupled climate model CSIRO Mk3L and ran a sensitivity study with NCAR CAM3. The climate modeling experiments presented here do not provide a full reconstruction of these past climates; rather they are designed as sensitivity experiments to assess the mechanism of obliquity forcing upon climate as proposed in this paper.

CSIRO Mk3L is designed for millennial scale climate simulations comprising a fully interacting ocean, atmosphere, land-surface and sea-ice components, and is designed for millennial scale climate simulations with an ocean model of 1.6**°** latitude x 2.8**°** longitude resolution and 21 vertical levels, and an atmospheric model with resolution of 3.2**°** latitude x 5.6**°** longitude and 18 pressure levels. The model includes flux adjustments, which correct the model state towards a modern-day climatology in the control simulations. CSIRO Mk3L has accurately simulated other paleoclimate scenarios, including the mid-Holocene at 6 ka for which there are extensive model and proxy data available for comparison[^14^](#_ENREF_14). We chose CSIRO Mk3L for its computational efficiency in millenial-scale simulations, while it retains full dynamic complexity in its sub-models. Land surface properties including vegetation, soil types and ice cover are designed to resemble those of the 21 ka climate, and are held fixed across the three paleoclimate experiments. Sea level changes are not included, and the land geometry is the same as a modern-day scenario. Thus the greenhouse gas and orbital forcing provide the only differences that drive each simulation toward its own distinct climate state. Each simulation is equilibrated in a time-slice experimental framework, with the forcing held fixed for 2000 years in each scenario. Each experiment was initiated from a modern-day climatology and spun up for 2,000 years using the forcing described in Table 1. The orbital forcings were taken from long-term insolation records[^15^](#_ENREF_15), and the greenhouse gas concentrations were taken from the Taylor Dome Ice Core, Antarctica The greenhouse gas concentrations are all implemented using the equivalent CO2 (CO2-e) radiative forcing shown in Table S2.

We further tested the mechanisms suggested in this study with the atmospheric component of the National Center for Atmospheric Research (NCAR) model, i.e. the Community Atmosphere Model (CAM3)[^16^](#_ENREF_16). The model was run in T42 horizontal resolution (approximately 2.8x2.8°), with 26 vertical levels in sigma‐pressure hybrid coordinates. The atmospheric model was forced by the two key factors that we hypothesize as the primary drivers of the eLGM. The resultant change in wind and temperature can be seen in Figure S1, with i) orbital parameters and greenhouse gases, and ii) northward expansion of Antarctic sea ice. In the first set of experiments, we force the model with the orbital parameters and greenhouse gases conditions for the 21ka and 28.5ka climate (Figure S1 a, b, c and d).  In the second set of experiments (ICE+), Antarctic sea‐ice was increased by 7° of latitude over its climatological position and compared to a present day run (Figure S1 d and e). Although these experiments neglect the ocean feedback, they allow us to examine the atmospheric response to each factor separately.

Table S1: Site locations, cosmogenic age constraints for Stage 2 glacial advances in Patagonia

Table 2 Greenhouse gas concentrations and orbital parameters of the four CSIRO Mk3L model simulations presented.

| Epoch | CO_2_ | N_2_O | CH_4_ | CO_2_-e | Obliquity | Eccentricity | Longitude |
| --- | --- | --- | --- | --- | --- | --- | --- |
|  | (ppm) | (ppb) | (ppb) | (ppm) |  |  | Perihelion |
| control | 280 | 270 | 760 | 280 | 23.446 | 0.016724 | 102.07 |
| 21ka | 185 | 200 | 350 | 167.1 | 22.949 | 0.018994 | 114.15 |
| 28.5ka | 200.4 | 211 | 367.7 | 183.1 | 22.207 | 0.016803 | 353.28 |
| 49ka | 199 | 224 | 464 | 186.5 | 24.435 | 0.01292 | 62.209 |

**Figure S1.** NCAR CCSM3 model simulations showing differences in simulated zonally averaged air temperature (a-b) and (c-d) zonal wind between the 21ka and 28.5ka climate. (e-f) Differences in the zonally averaged zonal wind between the experiments forced with a northward expansion of Antarctic sea-ice and the present day climatology. Top row: Warm season (January-March). Bottom row: Cold season (August-October). Units in Celsius and m/s, respectively (created in Adobe Illustrator CS5 v15.0.0).

1 Kaplan, M. R. *et al.* In-situ cosmogenic 10Be production rate at Lago Argentino, Patagonia: Implications for late-glacial climate chronology. *Earth and Planetary Science Letters* **309**, 21-32, doi:10.1016/j.epsl.2011.06.018 (2011).

2 Putnam, A. E. *et al.* In situ cosmogenic 10Be production-rate calibration from the Southern Alps, New Zealand. *Quaternary Geochronology* **5**, 392-409, doi:10.1016/j.quageo.2009.12.001 (2010).

3 Balco, G., Stone, J. O., Lifton, N. A. & Dunai, T. J. A complete and easily accessible means of calculating surface exposure ages or erosion rates from 10Be and 26Al measurements. *Quaternary Geochronology* **3**, 174-195, doi:10.1016/j.quageo.2007.12.001 (2008).

4 Nishiizumi, K. *et al.* Absolute calibration of 10Be AMS standards. *Nuclear Instruments and Methods in Physics Research Section B: Beam Interactions with Materials and Atoms* **258**, 403-413, doi:10.1016/j.nimb.2007.01.297 (2007).

5 Reimer, P. J. *et al.* IntCal09 and Marine09 radiocarbon age calibration curves, 0-50,000 years cal BP. *Radiocarbon* **51**, 1111-1150 (2009).

6 Denton, G. H., Lowell, T.V., Heusser, C.J., Schluchter, C., Andersen, B.G., Heusser, L.E., & Moreno, P. I., Marchant, D.R., Geomorphology, stratigraphy, and radiocarbon chronology of Llanquihue drift in the area of the southern Lake District, Seno Reloncavi, and Isla Grande de Chiloe, Chile. *Geogr. Ann. Ser. A Phys. Geogr.* **81A,**, 167–229. (1999).

7 McCulloch, R. D., Fogwill, C.J., Sugden, D.E., Bentley, M.J., Kubik, P.W. Chronology of the last glaciation in central Strait of Magellan and Bahia Inutil, southernmost South America. *Geogr. Ann. Ser. A Phys. Geogr.* **87A,**, 289–312. (2005).

8 Kaplan, M. R., Ackert, R.P., Singer, B. S., Douglass, D.C., Kurz, M.D. . Cosmogenic nuclide chronology of millennial-scale glacial advances during O-isotope Stage 2 in Patagonia. *Geological Society of America Bulletin* **116**, 308-321 (2004).

9 Hein, A. S. *et al.* The chronology of the Last Glacial Maximum and deglacial events in central Argentine Patagonia. *Quaternary Science Reviews* **29**, 1212-1227, doi:10.1016/j.quascirev.2010.01.020 (2010).

10 Kaplan, M. R. *et al.* Southern Patagonian glacial chronology for the Last Glacial period and implications for Southern Ocean climate. *Quaternary Science Reviews* **27**, 284-294, doi:10.1016/j.quascirev.2007.09.013 (2008).

11 Schaefer, J. M. *et al.* Near-synchronous interhemispheric termination of the last glacial maximum in mid-latitudes. *Science* **312**, 1510-1513, doi:10.1126/science.1122872 (2006).

12 Hallet, B. a. P., J. Surface Dating of Dynamic Landforms: Young Boulders on Aging Moraines. *Science* **265**, 937-940 (1994).

13 Phipps, S. J. The CSIRO Mk3L climate system model v1.2, 121 pp., Technical Report, Antarctic Climate and Ecosystems Cooperative Research Centre, Hobart, Tasmania, Australia. ( 2010).

14 Phipps, S. J. *et al.* The CSIRO Mk3L climate system model version 1.0 - Part 2: Response to external forcings, . *Geoscientific Model Development,* **5**, 649-682, (2012).

15 Berger, A. L. Long-Term Variations of Daily Insolation and Quaternary Climatic Changes. *Journal of the Atmospheric Sciences* **35**, 2362-2367 (1978).

16 Collins, W. D. & P. J. Rasch, e. a. Description of the NCAR Community Atmosphere Model (CAM 3.0), Technical Report NCAR/TN-464+STR,. *National Center for Atmospheric Research, Boulder, Colorado,*, 210 (2004).
